# Skin Telocytes Could Fundament the Cellular Mechanisms of Wound Healing in Platelet-Rich Plasma Administration

**DOI:** 10.3390/cells13161321

**Published:** 2024-08-08

**Authors:** Catalin G. Manole, Vlad M. Voiculescu, Cristina Soare, Laura Cristina Ceafalan, Mihaela Gherghiceanu, Mihail E. Hinescu

**Affiliations:** 1 Department of Cellular and Molecular Biology and Histology, “Carol Davila” University of Medicine and Pharmacy, 020021 Bucharest, Romania; 2 Ultrastructural Pathology Laboratory, “Victor Babeș” National Institute of Pathology, 050096 Bucharest, Romania; 3 Department of Oncological Dermatology, “Carol Davila” University of Medicine and Pharmacy, 020021 Bucharest, Romania; 4 Cell Biology, Neurosciences and Experimental Myology Laboratory, “Victor Babeș” National Institute of Pathology, 050096 Bucharest, Romania; 5 “Victor Babeș” National Institute of Pathology, 050096 Bucharest, Romania

**Keywords:** platelet-rich plasma (PRP), dermatology, regenerative dermatology, skin repair/remodeling, telocytes

## Abstract

For more than 40 years, autologous platelet concentrates have been used in clinical medicine. Since the first formula used, namely platelet-rich plasma (PRP), other platelet concentrates have been experimented with, including platelet-rich fibrin and concentrated growth factor. Platelet concentrates have three standard characteristics: they act as scaffolds, they serve as a source of growth factors and cytokines, and they contain live cells. PRP has become extensively used in regenerative medicine for the successful treatment of a variety of clinical (non-)dermatological conditions like alopecies, acne scars, skin burns, skin ulcers, muscle, cartilage, and bone repair, and as an adjuvant in post-surgery wound healing, with obvious benefits in terms of functionality and aesthetic recovery of affected tissues/organs. These indications were well documented, and a large amount of evidence has already been published supporting the efficacy of this method. The primordial principle behind minimally invasive PRP treatments is the usage of the patient’s own platelets. The benefits of the autologous transplantation of thrombocytes are significant, representing a fast and economic method that requires only basic equipment and training, and it is biocompatible, thus being a low risk for the patient (infection and immunological reactions can be virtually disregarded). Usually, the structural benefits of applying PRP are attributed to fibroblasts only, as they are considered the most numerous cell population within the interstitium. However, this apparent simplistic explanation is still eluding those different types of interstitial cells (distinct from fibroblasts) that are residing within stromal tissue, e.g., telocytes (TCs). Moreover, dermal TCs have an already documented potential in angiogenesis (extra-cutaneous, but also within skin), and their implication in skin recovery in a few dermatological conditions was attested and described ultrastructurally and immunophenotypically. Interestingly, PRP biochemically consists of a series of growth factors, cytokines, and other molecules, to which TCs have also proven to have a positive expression. Thus, it is attractive to hypothesize and to document any tissular collaboration between cutaneous administered PRP and local dermal TCs in skin recovery/repair/regeneration. Therefore, TCs could be perceived as the missing link necessary to provide a solid explanation of the good results achieved by administering PRP in skin-repairing processes.

## 1. Introduction

**Figure 1:**
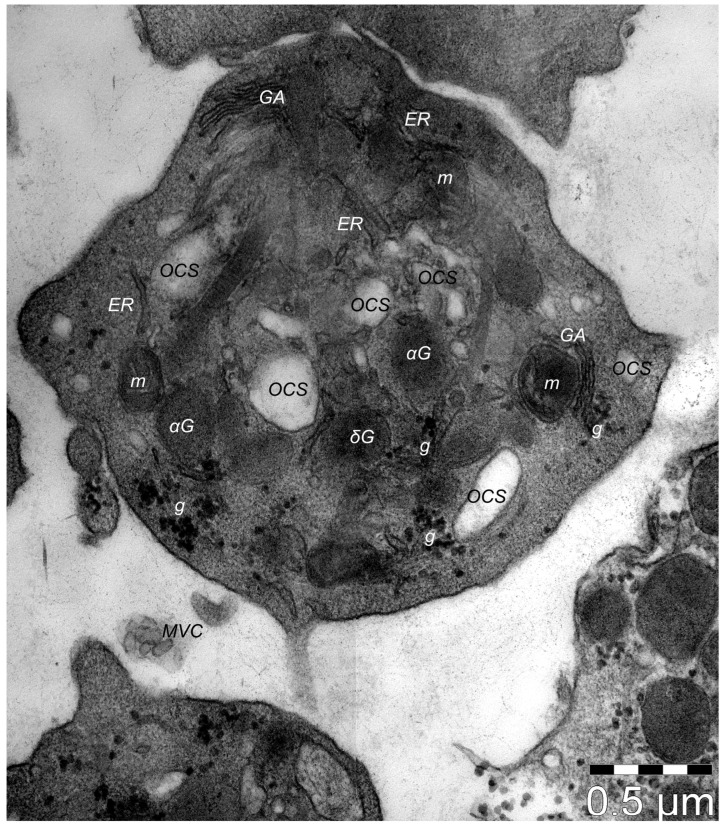
Platelet. Transmission electron microscopy. The platelet cell membrane is encompassing an aliquot of cytoplasm of the thrombocytogenic megakaryocyte, in which different organelles that can be visible (e.g., the Golgi apparatus, GA, mitochondria, m, endoplasmic reticulum, ER), but also the open canalicular system (OCS), alpha granules (αG) and dense granules (δG). The lisosomes with a bundle-like appearance are disposed among the elements of OCS. Randomly dispersed glycogen granules (g) can be observed.

Platelets have fundamental roles in homeostasis and thrombosis. This is evident in tissue injury caused by trauma or ischemia, like in lesions produced by myocardial infarction or stroke, when the coagulation system and immune responses activate very early, initiating the healing process. Platelets (Figure 1) are fragments of thrombocytogenic megakaryocytes that are among the first cells that penetrate and accumulate within the lesioned tissue and, when activated, release a multitude of biologically active mediators into their microenvironment [1]. Apart from these traditional roles, in recent years, new and important ones have surfaced. Thrombocytes, through their growth factors (GFs), cytokines, antibiotic peptides, and extracellular matrix modulators are involved in a cascade of events starting with angiogenesis in damaged tissue, stabilizing blood vessels and restoration of connective tissue, the transformation of mesenchymal stem cells into fibroblasts, etc.

Also, more evidence suggests that platelets participate in regulating the balance between cell proliferation and cell apoptosis, the latter having a crucial role in immune tolerance with important implications in the correct regeneration of damaged tissue [2,3,4,5]. The recruitment, proliferation, and activation of smooth muscle cells (SMCs), mesenchymal stem cells (MSCs), and other cell types that participate in tissue regeneration are controlled by cytokines, chemokines, and growth factors released from the platelets. Pro-apoptotic mediators such as CD40 ligand (CD40L), Fas ligand (Fas L), tumor-necrosis-factor (TNF)-related apoptosis-inducing ligand (TRAIL), TNF-like weak inducer of apoptosis (TWEAK), and LIGHT (a member of the tumor necrosis factor superfamily), and antiapoptotic agents like hepatocyte growth factor (HGF), stromal-cell-derived factor-1 (SDF-1), serotonin, adenosine diphosphate, and sphingosine-1-phosphate are released from platelets, by which they modulate the balance between apoptosis and cell survival. In addition, platelet-derived microparticles (MPs) can induce apoptosis in endothelial cells (ECs) and SMCs and can provide survival signals to monocytes and stem cells [6]. All these data are relevant and may support the explanation of the complex tissue repair mechanisms that platelets are controlling. Granzyme B is also a mediator of platelet-induced apoptosis in the spleen and lungs. High-mobility group box 1 (HMGB1), a nuclear non-histonic protein, is a danger signal that is exported to the cell surface from platelets for activation and regulates apoptosis and autophagy in tumor cells (TCs), depending on its redox state. Thus, platelets are involved in the control of complex tissue repair mechanisms [6].

Moreover, in response to vascular injury, platelets become activated, secondarily releasing their granular content, amplifying the recruitment and activation of additional platelets, stabilizing clot formation, and initiating tissue repair processes. Prostaglandin E2 (PGE2), an important bioactive lipid produced by platelets and other cells during inflammatory responses, participates in the orchestration of vasodilation, which increases blood flow to the affected area, and the modulation of inflammation [7]. On the other hand, it has been shown that among endothelial cells, stimulated platelets are able to produce nitric oxide (NO), which is a limited inhibitor of platelet activation, but by reducing calcium levels, which is a major inhibitor of platelet recruitment [8]. Additionally, NO is a smooth muscle relaxant involved in vasodilatation and blood flow regulation [9],

We recently scrutinized the literature to assess the acknowledged mechanisms of tissue repair and remodeling induced by platelets administered through PRP. We considered not only the classical known mechanism orchestrated by cytokines, synthesized by growth factors, and secreted by platelets, but also a potential novel mechanism that implies the presumptive activation and recruitment of dermal telocytes (TCs)—a distinct type of interstitial cell with roles in tissue regeneration. TCs distinctiveness resides in their particular (ultra)structural features, their microRNA expression, and their proteomics and secretomics [10].

At the skin level, TCs are found in both the papillary and reticular dermis. This has been evidenced using various techniques. A transmission electron microscopy (TEM) investigation of the human dermis found TCs in constant spatial relations with vascular structures, immune cells, and/or adnexal structures [11]. However, the most particular structural feature of TCs is their unique structure combined with their particular cytokine profile [12]. TCs are still to be fully characterized and their roles within the interstitium to be fully understood, but they seem to be involved in tissue remodeling and regeneration, mainly by their pro-angiogenic potential.

## 2. PRP Plasma Biochemistry and Active Factors

The granular cargo of platelets is different in platelets’ granules. α-granules contain both membrane-bound proteins (usually expressed on the platelet membrane) and soluble proteins (which are secreted into the extra-platelet space) [13,14,15]. The platelets’ integral membrane proteins are mainly represented by most of the glycoproteins found in other cells. These glycoproteins are involved in platelet aggregation and adhesion, in thrombin generation, or in protein endocytosis [16,17,18].

## 3. The Composition of the Platelets’ α-Granules

The Golgi apparatus (specifically, the trans-Golgi network) represents the origin of α-granules. Thus, they have a secretory phenotype similar to late endosomes and lysosomes and are considered lysosome-related organelles similar to basophil granules, azurophil granules, melanosomes, Weibel–Palade granules, Odland bodies in keratinocytes, and lamellar bodies in type I pneumocytes [19]. Usually, they demonstrate similarities with these organelles, like endosomal filiation, the acid content due to the presence of various enzymes, and the protein composition of the lysosome membranes [20]. They develop from the former small granular precursors found within the cytoplasm of thrombocytogenic megakaryoblasts (MKs), which maturate α-granules and deliver them, by thrombopoiesis, to platelets for final packaging. The organelles that originate α-granules either accumulate different compounds via synthetic routes (e.g., von Willebrand Factor, β-thromboglobulin, or platelet factor 4—PF4), or acquire them by endocytosis, (e.g., albumin, fibrinogen, or immunoglobulin G) [21]. In terms of morphology, ultrastructural studies have indicated a few different types of α-granules, including tubular, spherical, and multivesicular subtypes [22]. The matured granules are released from the platelets in different pathological processes of hemostasis and wound repair, inflammation, tumor metastasis, or angiogenesis [23]. The dynamics of platelet secretion indicates their roles and functions, and their performant action in all processes they are involved in. Thus, we presume that the number, sequence, and amount of each of these factors that are released from platelets are orchestrating the steps of tissue regeneration/repair in terms cellular and molecular phenomena that feature in the healing process.

The platelets’ α-granules contain growth factors, chemokines, adhesion molecules, integral membrane proteins, coagulants, anticoagulants, and other molecules (summarized in Table 1).

### 3.1. Growth Factors

Growth factors, which are involved in regulating different cellular processes like cell proliferation, differentiation, and maturation, typically act as signaling molecules.

#### 3.1.1. Platelet-Derived Growth Factor (PDGF), AA-AB-BB

The AA, AB, and BB are isoforms of PDGF and have been extensively studied in striated skeletal muscle cells. They have proven to have similar functions, stimulating osteoblast replication and DNA synthesis, and to have mitogenic activity (mainly observed in periosteum). However, in terms of functionality, PDGF BB is more potent than the AA and AB isoforms [24]. Thus, PDGF could promote the maintenance of and increase in the cellular pool of the osteoblastic lineage, being mitogenic for mesenchymal cells and osteoblasts, and perhaps explain the efficiency of PRP treatments in orthopedic pathology, where such treatments have proven to have noticeable benefits. Moreover, PDGF stimulates chemotaxis and mitogenesis in fibroblasts, glial cells, and smooth muscle cells [25], but also stimulates macrophage and neutrophil chemotaxis [26]. PDGF AB regulates collagenase secretion and collagen synthesis [27], through boosting the tissue regeneration/remodeling that are featured in (skin) PRP treatments. PDGF is found in platelets, in endothelial cells, macrophages, and smooth muscle cells [25].

#### 3.1.2. Transforming Growth Factor β (TGFβ)

Transforming growth factor β (TGFβ) is a highly basic protein acting as a potent inhibitor for different cell types. TGFβ is contained by platelets’ α-granules but is also an extracellular matrix protein [28]. The TGFβ1 isoform is specific to human platelets and is found either complexed with both latent TGFβ-binding protein (LTBP) and the latency-associated peptide (LAP) or complexed only with LAP (without LTBP) [29,30]. TGFβ can be found in macrophages, T-lymphocytes, or keratinocytes [25]. TGFβ, through its four fasciclin1 domains, is involved in cellular proliferation, migration, adhesion, and differentiation, being implicated as a ligand for several integrins, collagen I, II, and IV, laminin, fibronectin, and glycosaminoglycan [31,32]. It was previously shown that the interaction between platelets and tumor cells helps promote the invasiveness and malignancy of the latter [33]. However, TGFβ inhibits the cell growth and proliferation of normal and malignant cells (including hematopoietic cells, macrophages, lymphocytes, and megakaryocytopoiesi) [25,34]. Moreover, TGFβ stimulates the proliferation of undifferentiated mesenchymal cells [35], regulating the endothelial-to-mesenchymal transition [36], or fibroblastic and osteoblastic mitogenesis [37] by mitogen-activated protein kinase (MAPK) pathway. Thus, TGFβ could act as a regulator of angiogenesis induced by PRP administration, maintaining an equilibrium between the old and newly formed endothelial cells. On the other hand, by the antagonistic effects on VEGF, TGFβ1 induces the rapid apoptosis of endothelial cells [38]. However, the inhibition of VEGF blocks angiogenesis and/or apoptosis induced by TGFβ1 [39]. Moreover, the TGFβ/Smad signaling pathway regulates collagen synthesis and its deposition into the interstitial structure [40].

#### 3.1.3. Vascular Endothelial Growth Factor (VEGF)

Vascular endothelial growth factor (VEGF) is a homodimeric protein that is produced either by normal cells (e.g., platelets, leukocytes, macrophages, keratinocytes, or endothelial cells) or by tumor cells (which can synthesize great amounts of VEGF). The tumoral cells’ production of VEGF alters its plasmatic concentration levels, and thus VEGF serum levels could be predictive of cancer prognosis. However, VEGF is basically a key regulator of angiogenesis, since it stimulates neo-angiogenesis, contributing to an increased vascular permeability, as well as promoting endothelial cell viability, migration, and differentiation [41,42,43]. VEGF is normally released after the activation of thrombin by platelets. However, in many tumoral contexts, leukocytes are a more important source of VEGF, but in this case the newly formed tumoral blood vessels display many structural and functional aberrations [44,45]. Considering the positive expression of TCs for VEGF, it would be interesting to more deeply explore the structural outcomes regarding dermal TCs (or of stromal TCs with other locations) after administering PRP.

#### 3.1.4. Epidermal Growth Factor (EGF)

Epidermal growth factor (EGF) is a small protein (of about 6000 Da) that is involved in cell development and differentiation [46]. EGF is synthesized by different cells (platelets, macrophages, and monocytes). Moreover, the cells of the human submaxillary glands and kidneys are the main sources of EGF (other secondary sources being epithelial cells of the duodenal Brunner glands and placenta). EGF is also present in body fluids like saliva, urine, and plasma [25,47]. After binding to its ligand and increasing the intracellular tyrosine kinase activity, thereby activating the signaling cascade and promoting the cell proliferation of keratinocytes, fibroblasts, endothelial cells, etc., angiogenesis also acts as a downregulator of apoptosis [48].

#### 3.1.5. Fibroblast Growth Factor (FGF)

Fibroblast growth factor (FGF) is a signaling protein produced mainly by macrophages (but in smaller amounts it is also produced by platelets, mesenchymal cells, osteoblasts, and chondrocytes) [49]. FGF promotes endothelial cell proliferation, and along with other pro-angiogenic factors (e.g., VEGF), is involved in (neo-)angiogenesis, thus having potential biological functions in tissue repair/regeneration [49,50]. By connecting to the plasma membrane’s FGF receptors, it mainly activates the RAS/MAP kinase pathway, but also other intercellular signaling pathways, like the PI3K/AKT pathway and PLCγ pathway [51]. The published data on FGF indicate its involvement in modulating biological functions such as cell proliferation, survival, migration, and differentiation [52]. It is interesting the synergic effect this growth factor has (along with VEGF, EGF, and other pro-angiogenic molecules), perhaps in conjunction with the pro-angiogenic potential of other stromal cells (including TCs).

#### 3.1.6. Connective Tissue Growth Factor (CTGF)

Connective tissue growth factor (CTGF) is a non-structural small protein (of 38,000 Da) that is dynamically expressed within the extracellular matrix and has signaling and regulatory implications. Platelets and fibroblasts contain CTGF and can release this protein after their activation. The current published data indicate an increased expression of CTGF in atherosclerotic blood vessels, and the level of expression is directly proportional to shear stress [50,53], but CTGF also has increased positive expressions in fibrotic disorders [15]. However, large normal blood vessels and developing blood vessels highly express CTGF mRNA, suggesting its involvement in angiogenesis and blood vessel structural maintenance processes [54]. CTGF has been shown to have roles in skeletogenesis [55], cell adhesion, proliferation, and wound repair [56]. Thus, CTGF could be involved in the structural recovery of the regressing interstitium.

#### 3.1.7. Insulin-like Growth Factor (IGF-1)

Insulin-like growth factor (IGF-1) is a peptide hormone with roles in cell proliferation, differentiation, and migration, and is a typical constituent of platelets’ α-granules. Moreover, platelets express the IGF-1 receptor on their membrane, and it is assumed to be involved in the onset and amplification of platelets’ response to acute cardiovascular diseases [57]. IGF-1 is also secreted by the liver under the influence of growth hormone [58] and is secreted at the level of chondroblasts acting locally as a paracrine hormone [59].

#### 3.1.8. Hepatocyte Growth Factor (HGF)

Hepatocyte growth factor (HGF) is a multifunctional cytokine involved in various biological mechanisms including cell proliferation, migration, and cell survival [60]. HGF is synthesized as a pro-HGF precursor by mesenchymal cells, and circulates within the serum, but can also be found within the connective tissue matrix, strongly bound to heparin sulfate [61]. Human platelets have a positive expression for HGFR on their membrane; thus, HGF acts as an inhibitor for platelets with some benefits in thromboses [62]. HGF is involved in endothelial/epithelial cell growth and mobility, as well as in morphogenesis and regeneration/repair skin processes through angiogenesis [63,64]. Its functions at least partially explain some of the regeneration/repair assets after PRP administration.

#### 3.1.9. Keratinocyte Growth Factor/Fibroblast Growth Factor-7 (KGF/FGF-7)

Keratinocyte growth factor/fibroblast growth factor-7 (KGF/FGF-7) is a small proteic mitogen (28 kDa) produced by mesenchymal cells (e.g., fibroblasts) that acts as a protector for several types of epithelial cells (keratinocytes, enterocytes, and hepatocytes), also regulating their proliferation, migration, and differentiation [65,66]. KGF has also proven to exhibit reparatory capacities in lung epithelia, expressing protective and restoring capacities after radiotherapy thymic damage [67]. KGF stimulates the cells involved in angiogenesis, and by epithelial fibroblast growth factor receptor 2 (FGFR2) mediates epithelial–mesenchymal interactions [68]. However, FGFR2 is a tyrosine kinase receptor that is also required to bind with co-receptors (proteoglycans) for signal transduction [69]. KGF and other FGFs have mitogenic and motogenic properties, either in vivo or in vitro, but KGF is more potent and overexpressed in the early stages of wound healing [70]. Cytokines (IL-1 and IL-6), growth factors (PDGFβα, TGFα, and HGF) and hormones (steroid hormones, estrogen, progesterone, glucocorticoids, and androgens) regulate the expression of FGF7 in different tissues [68].

#### 3.1.10. Angiopoietin-1 (ANG-1)

Angiopoietin-1 (ANG-1) is an angiogenic glycoprotein, along with angiopoietin-2, angiopoietin-3, and angiopoietin-4, all of which are members of the angiopoietin family [71]. ANG-1, together with VEGF, is involved in endothelium inflammation and blood vessel integrity [72]. ANG-1 has affinity for Tie-2 receptors (found on endothelial cells), by which ANG-1 influences the late stages of angiogenesis, but is also involved in the remodeling and stabilization of older blood vessels [73]. The tandem ANG-1/Tie-2 has roles in the activation and recruitment of pericytes to stabilize old blood vessels [74]. In addition to VEGF, platelet α-granules also contain ANG-1, the number of platelets in fact influencing the plasmatic concentration of both vascular active proteins [75]. ANG-1 is an important mediator of angiogenesis, maintaining endothelial cell integrity and having anti-inflammatory functions [76].

### 3.2. Chemokines

#### 3.2.1. Growth-Regulated Protein Alpha (CXCL1/GRO α/KC/CINC-1)

Growth-regulated protein alpha (CXCL1/GRO α/KC/CINC-1) is a chemokine with angiogenic functions that interacts with CXCL2 and attracts neutrophils. Inter alia, the activity of CXCL1 is involved in the regulation of platelet function and is associated with tumoral angiogenesis [77]. Platelets in myeloproliferative neoplasms have an increased expression of CXCL1 associated with an elevated GRO-α production by CD56+/CD14+ monocytes, apparently with implications in disease progression [78].

#### 3.2.2. Platelet Factor 4/Chemokine (C-X-C Motif) Ligand 4 (PF4/CXCL4)

Platelet factor 4/chemokine (C-X-C motif) ligand 4 (PF4/CXCL4), the first discovered chemokine, is a positively charged tetrameric chemokine protein belonging to a larger family of CXC chemokines [79]. PF4 produced by MKs is stored in α-granules and further transferred to platelets, which then release it after their activation [80]. However, PF4 can also be synthesized by monocytes [81]. Physiologically, PF4 promotes blood clotting, with roles in hematopoiesis, angiogenesis, hemostasis, and thrombosis. Moreover, PF4 has clear proinflammatory properties and has implications in innate/adaptive immunity [82,83,84]. However, PF4 is an important mediator of heparin-induced thrombocytopenia, a post-thrombotic side effect of a few therapies [79]. Usually, PF4 is involved in complexes with polyanion heparin and after binding to bacteria, PF4 induces specific antibodies that enhance bacterial phagocytosis [85].

#### 3.2.3. Epithelial-Cell-Derived Neutrophil-Activating Peptide-78 (ENA-78)/C-X-C Motif Chemokine 5 (CXCL5)

Epithelial-cell-derived neutrophil-activating peptide-78 (ENA-78)/C-X-C motif chemokine 5 (CXCL5) is a potent neutrophil chemotactic molecule also found in the platelets’ α-granules. Initially, it was presumed to be exclusively synthesized by epithelial cells [86]. It binds to G-protein-coupled receptor CXCL2, leading to neutrophilic recruiting. It is also involved in angiogenesis and the development of capillary networks within lesioned tissue. Thus, it could represent a critical factor in tissue repair and regeneration or have major implications in connective tissue remodeling [87]. The involvement of CXCL5 in the pathogenesis and progression of inflammatory disorders was previously documented. This small chemotactic protein may contribute to repair and neovascularization processes and could represent a potential molecular target in regenerative medicine [88].

#### 3.2.4. C-X-C Motif Chemokine 7 (CXCL7)/Neutrophil-Activating Peptide 2 (NAP-2)

C-X-C motif chemokine 7 (CXCL7)/neutrophil-activating peptide 2 (NAP-2) regulates neutrophil recruitment after vascular injury, by activating CXC chemokine receptor 2 (CXCR2) and binding to sulfated glycosaminoglycans (GAGs) [89]. Additionally, the interaction with GAGs dictates the affinity of CXCL7 for CXCR2, and therefore it regulates the migration of neutrophils within the thrombus, promoting tissue repair and minimizing tissue damage and disease [90].

#### 3.2.5. C-X-C Motif Chemokine 8 (CXCL8)/Interleukin-8 (IL-8)

C-X-C motif chemokine 8 (CXCL8)/interleukin-8 (IL-8) plays definitory roles in tissue response to injury, being the most important neutrophil-attracting and activating chemokine [91]. It interacts with CXCR1, CXCR2, and GAGs, being involved in regulating the cell adhesion, chemoattraction, and activation of leukocytes [92]. On the other hand, CXCL8 may possess angiogenic properties by stimulating the proliferation of endothelial cells and/or the endothelial-to-mesenchymal transition, leading to the formation of new blood vessels, or cell migration to damaged tissue, thus favoring the entire process of tissue repair/regeneration [93].

#### 3.2.6. Stromal-Cell-Derived Factor 1 α/C-X-C Motif Chemokine 12 (SDF-1α/CXCL12)

Stromal-cell-derived factor 1 α/C-X-C motif chemokine 12 (SDF-1α/CXCL12) is a chemokine expressed by fibroblasts, stromal cells, and endothelial cells, but also found within platelets’ α-granules. SDF-1α is a strong chemotactic cytokine for leukocytes, hematopoietic progenitor cells, that provides angiogenic support through its involvement in endothelial cell proliferation [25,94]. Actually, SDF-1α is a growth stimulator of pre-B lymphocytes and a chemoattractant for T-lymphocytes, monocytes, and hematopoietic stem cells [95]. Platelet-derived SDF-1α is involved in stem cell adhesion phenomena, also having implications in the differentiation of CD34+ cells into endothelial cells [96,97]. In cell culture, SDF-1α combined with thrombopoietin was demonstrated to favor the development of CD34+ cells [98]. Moreover, SDF-1α expression is involved in stem cell recruitment and homing after myocardial infarction, in cardiomyocytes death, and in scar remodeling [99]. Apparently, SDF-1α and PRP demonstrate synergy in bone regeneration/repair by promoting angiogenesis [98], this association having promising results for in situ regenerative/repair therapies.

#### 3.2.7. Monocyte Chemoattractant Protein (MCP-1)/Chemokine C-C Ligand-2 (CCL2)

Monocyte chemoattractant protein (MCP-1)/Chemokine C-C ligand-2 (CCL2) is involved in chemotaxis of immune and inflammatory cells including monocytes, migration of monocytes and their transforming into macrophages [100]. In breast cancer CCL2 acts as a promoter of metastasis [101]. CCL2 also represents a proinflammatory cytokine that promotes the accumulation of inflammatory infiltrates and has immunomodulatory effects, stimulating the expression of adhesion molecules in monocytes, and stimulating the secretion of other such cytokines.

#### 3.2.8. Macrophage Inflammatory Protein-1α (MIP-1α)/Chemokine C-C Ligand-3 (CCL3)

Macrophage inflammatory protein-1α (MIP-1α)/Chemokine C-C ligand-3 (CCL3) is involved in pathogenesis of a few inflammatory conditions such as multiple myeloma and rheumatoid arthritis. MIP-1α could also have diagnostic potential in these diseases and gained its applicative potential as biomarker [102]. CCL3 could have important roles in the progression of hematopoietic malignancies [103].

#### 3.2.9. Chemokine C-C Ligand-5 (CCL5)/Regulated on Activation, Normal T-Cell Expressed and Secreted (RANTES)

Chemokine C-C ligand-5 (CCL5)/Regulated on Activation, Normal T-cell Expressed and Secreted (RANTES) is a proinflammatory chemokine secreted by platelets and intensely involved in immunoregulatory and inflammatory processes by recruiting immune cells to inflammation sites. CCL5 is ligating the P-selectin and thus is favoring the development of monocyte–macrophage inflammatory infiltration [104]. Usually CCL5/RANTES aggregates can be found in the presence of cell surface GAGs [105].

### 3.3. Adhesion Molecules

#### 3.3.1. Fibrinogen

Immune electron microscopy and cell fracturing studies indicated the presence of fibrinogen within α-granules. However, only 3% of circulating fibrinogen is stored in platelets [106]. Platelet fibrinogen is provided by circulating plasma, but fibrinogen uptake could occur either in platelets or MKs [107]. Previous published studies documented fibrinogen uptake by platelets after plasmatic fibrinogen infusion. On the other hand, MKs can also incorporate fibrinogen into α-granules [22]. However, within the α-granule matrix, fibrinogen (together with other active factors like TNFα, TGFβ, and PF4) is uniformly distributed and frequently colocalized with VAMP8 [108,109].

#### 3.3.2. Thrombospondin

Thrombospondin is a modular glycoprotein responsible for different functions of platelets and other cells. Thrombospondin is produced by MKs and is stored in α-granules, having important roles in aggregation, agglutination, the adhesion of platelets, and facilitating cell proliferation and motility [110]. At vascular injury sites, thrombospondin regulates hemostasis by the modulation of cyclic adenosine monophosphate (cAMP) [111].

#### 3.3.3. The von Willebrand Factor (vWF)

The von Willebrand factor (vWF) circulates either in plasma (being synthesized by endothelial cells) or is deposited within the α-granules of platelets (which are synthesized by MKs). The vWF bridges platelets and acts as a pro-aggregant factor [112]. Supplementarily, the vWF carries and protects coagulation factor VIII from degradation. Moreover, the vWF is involved in acute thrombolysis in blocked blood vessels, thus mitigating the damage produced by ischemia/reperfusion injury in infarcted tissue areas [113].

### 3.4. Integral Membrane Proteins

#### 3.4.1. Integrin αIIbβ3

Integrin αIIbβ3 is a signaling transmembrane glycoprotein that transmits information through platelet plasma, being highly expressed on the membrane of resting platelets [114], and in the open canalicular system and the α-granules [115]. Integrin αIIbβ3 has roles in tumor metastasis, platelet aggregation, hemostasis, and thrombosis. After activation, αIIbβ3 will ligate fibrinogen, fibrin, or the von Willebrand factor. The presence of this activated integrin represents a sign of platelet responsiveness [116].

#### 3.4.2. Glycoprotein (GP)Ib-IX-V Complex

Glycoprotein (GP)Ib-IX-V complex is in fact the receptor for the von Willebrand factor, present on the plasma membrane of the platelets (also found in MKs). Dysfunctional or absent complexes result in macrothrombocytopenia from Bernard–Soulier syndrome, a congenital bleeding disorder [117]. This integrin regulates the adhesivity of monocytes, thrombin activity, and the polarization of macrophages [118].

#### 3.4.3. Triggering Receptor Expressed on Myeloid Cells (TREM)-like Transcript 1 (TLT-1)

Triggering receptor expressed on myeloid cells (TREM)-like transcript 1 (TLT-1) belongs to a type I class of transmembrane proteins involved in adaptive immune responses. TLT-1 is exclusively expressed in MKs, which deliver it to the interior of platelet α-granules. Usually, it is transported onto the plasma membrane after platelet activation [119]. TLT-1 plays roles in platelet aggregation and hemostasis and uniquely acts in inflammatory bleeding [120].

#### 3.4.4. P-Selectin

P-selectin is a platelet-specific transmembrane protein located on the membrane of α-granules and can also be found in the Weibel–Palade granules of endothelial cells [121,122]. After platelet activation, P-selectin is transferred to the platelet plasma membrane where it acts as a recruiter of leukocytes (in injured blood vessels), but also acts as a receptor for neutrophils and monocytes [123]. The susceptibility to form platelet tumor cell microthrombi is reduced when P-selectin is absent [124]. P-selectin also participates in platelet recruitment and aggregation by promoting the bonds between platelets and fibrin or between multiple platelets at the site of endothelial injury. It also plays a role in the recruitment of leukocytes [125,126,127].

### 3.5. Coagulants and Anticoagulants

#### 3.5.1. Platelet Factor V

Platelet factor V is activated by thrombin and binds to activated platelets. It is mostly produced by liver MKs [128]. It plays dual roles in hemostasis, having both procoagulant and anticoagulant properties. The normal function of factor V contributes to the regulation of the coagulation cascade. It plays roles in platelet activation and could be a potential therapeutic target to prevent arterial thrombosis [129].

#### 3.5.2. Factor IX (Christmas Factor)

Factor IX (Christmas factor) is a serine protease belonging to the coagulation system produced in the liver. Post-translational changes modify the factor, which is secreted into circulation [130]. It determines the rate of thrombin formation [131].

#### 3.5.3. Factor XIII

Factor XIII is represented by a transglutaminase, which cross-links the fibrin network. It is activated by the thrombin cleavage and stabilizes the organization of the blood clot [132]. Moreover, it is involved in the further protection of the blood clot being degraded in the coagulation cascade [133]. Factor XIII has significant roles in angiogenesis and wound healing. Additionally, in bone tissue it is responsible for matrix stabilization [134].

#### 3.5.4. Growth Arrest Specific-6 (Gas6)

Growth arrest specific-6 (Gas6) is a protein that belongs to the vitamin-K-dependent protein family, and it has a structural resemblance to the anticoagulant of protein S, but it shows no anticoagulant functions [135]. Gas6 binds to tyrosine kinase receptors (Tyro3, Axl, and Mer) which activate the intracellular PI3K and Akt pathways. Thus, losing Gas6 functionality (by reducing platelet granule secretion) affects platelet aggregates, and blocking its receptor Gas6-R–αIIbβ3 integrin reduces thrombotic events [136]. Previous published data indicated the involvement of Gas6 in the etiology of various diseases (e.g., rheumatoid arthritis, breast cancer, etc.) [137].

### 3.6. Antimicrobial Agents

Beyond the well-acknowledged platelet function in vascular integrity and functionality, there is also an important body of published data indicating platelets’ active molecular response to microorganisms. These molecules fall into the groups of kinocidins, represented by the antimicrobial chemokines thrombocidin-1 (TC-1) and thrombocidin-2 (TC-2), and cationic host defense peptides (CHDPs), represented by the defensins and cathelicidins that are initially synthesized as immature peptides [138]. This antimicrobial capacity was experimentally tested against bacteria and fungi, and a structural and functional similarity between granulocytes and platelets confirmed the former results. The thrombin-activated platelets secrete platelet factor 4 (PF4) and connective-tissue-activating peptide 3 (CTAP-3), which act synergically in the platelet’s antibacterial capacity [139]. On the other hand, platelets recognize microorganisms by the damage-associated molecular patterns (DAMPs) they produce. DAMPs interfere with different platelet receptors like toll-like receptors (TLRs), among them TLR2 and TLR4 being the best described C-type lectin receptors, hemostatic platelet receptors GP Ib (glycoprotein Ib), and NLRs (NOD [nucleotide-binding oligomerization domain]-like receptors) [140].

### 3.7. Inflammatory/Immune Agents

There is progressively increasing evidence that platelets play crucial roles in the regulation of immunity and inflammatory responses. Platelets express in α-granules immune mediators like complement precursors C3, C4, C1 inhibitor, immunoglobulin G, etc. [141]. Platelets can bind pathogens like bacteria through synthesizing IgG to opsonize *E. Coli* [142], or through P-selectin, which ligates *S. aureus* [143], etc.; a large variety of viruses, including enteroviruses, influenza A viruses, coronaviruses, human immunodeficiency virus type-1 (HIV-1), herpes simplex virus type 1, etc. [144]; and parasites, contributing to the regulation of parasitemia, by binding to Plasmodium parasites species, thereby contributing to the intracellular accumulation of PF4 [145]. Moreover, TNFα, a proinflammatory molecule, can activate tissular pro-coagulant molecules, which can hyper-activate platelets through the endothelial cell’s molecular expression of anticoagulant molecules [146]. However, it will be interesting to document the interaction between pharmaceutical agents like non-steroidal anti-inflammatory drugs (NSAIDs) and PRP administration, in terms of the potential alteration of the PRP secretome induced by NSAIDs. Presently, it is well known that NSAIDs enhance TNFα production and its roles, presumably also in injected PRP [25].

## 4. Composition of Platelets’ Dense δ-Granules

Dense granules are electron-dense spherical vesicles of about 200 nm and are the second most abundant platelet organelle. δ-granules contain molecules involved in both homeostasis (evidenced by the bleeding propensity, which features in a few hematologic diseases—e.g., Chediak–Higashi syndrome, which exhibits a deficit of platelets’ dense granules) and thrombosis through the inhibition of arterial thrombosis by ADP, etc. Platelets’ δ-granules are maturated within MKs and then transferred to enucleated cells. The early endosomes contribute to the formation of δ-granules [147] and they also share lysosomal features; thus, dense granules are considered lysosome-related organelles, having and acidic content and housing lysosomal enzymes (e.g., tetraspanin CD63) [148].

These dense granules are small secretory deposits within platelets that harbor small molecules necessary for effective hemostasis (Table 2).

### 4.1. Nucleotides

#### 4.1.1. Adenosine Triphosphate (ATP)

The secretion of ATP (together with ADP) is a prerogative for platelet aggregation. ATP promotes the interaction between platelets, contributing to thrombus formation [149]. ATP, via P2X1 receptor, can activate platelets and inhibit the ADP platelet activation pathway, increasing the influx of Ca^2+^ within platelets [150].

#### 4.1.2. Adenosine Diphosphate (ADP)

Adenosine diphosphate (ADP) is usually contained by dense δ-granules serving as the primary source of ADP, since ADP is credited as a platelet aggregation factor, having multiple roles in hemostasis and thrombosis [151,152]. Platelets present plasma membrane P2Y receptors (P2Y1 and P2Y12) for ADP that are responsible for partial or full platelet aggregation. Another receptor, P2X1, which is activated by ATP, is responsible for the rapid influx of Ca^2+^ and produces platelet shape modification or platelet collagen activation by shear conditions [153]. During platelet activation, the granules containing ADP move towards the center of the cell and deliver ADP to the open canalicular system [154]. Other previous published data indicated ADP as an influential factor that regulates the release of VEGF from platelets’ α-granules. Moreover, it seems that ADP regulates the preferential release of other angiogenic promoters from platelets [155].

#### 4.1.3. Cyclic Adenosine Monophosphate (cAMP)

Cyclic adenosine monophosphate (cAMP) is a small hydrophilic molecule that acts as a second messenger within cells, being involved in cellular biological processes through intracellular signaling induction. cAMP is an important inhibitor of the platelet activation pathway [156].

#### 4.1.4. Uridine Triphosphate (UTP)

Uridine triphosphate (UTP), stored within the δ-granules of platelets, may antagonize P2Y12 receptor binding and, through cAMP, is involved in platelet aggregation. UTP could also antagonize collagen-induced platelet aggregation [157].

#### 4.1.5. Guanosine-5′-Triphosphate (GTP)

Guanosine-5′-triphosphate (GTP), found in δ-granules, participates in platelet activation and could be involved in eliciting platelet degranulation [158]. There are similarities between the thrombin molecule and the molecule of GTP. Considering this, it was hypothesized that GTP could be involved (in permeabilized platelets) in diacylglycerol formation that, through 1,4,5-inositol triphosphate, stimulates Ca^2+^ entry into platelets [159].

### 4.2. Bioactive Amines

#### 4.2.1. Serotonin (5-hydroxytryptamine)

Serotonin (5-hydroxytryptamine) is a biogenic amine with a broad spectrum of functions. Platelets transport and store significant concentrations of serotonin within δ-granules. By storing serotonin, platelets regulate and maintain the low concentration of plasma serotonin [160]. Abnormal concentrations of plasma serotonin can be found in sepsis, thrombosis, and myocardial infarction [161]. Serotonin is implicated in platelet development, especially in increasing their procoagulant activity by interacting with tissue factor, with significant involvement in thrombus formation [162,163]. On the other hand, many previously published data cited serotonin as an efficient vasoconstriction factor, mainly acting on venules and large blood vessels, possibly due to direct action on smooth muscle, the amplification of the muscle constriction mechanism, or the stimulation of the secretion of endogenous vasoconstrictors [163].

#### 4.2.2. Histamine

Histamine is stored in dense granules and is released, together with many other newly generated molecules, by thrombin activation. The platelet secretion of histamine could be regulated by immunological or aggreging factors and can explain some clinical findings in allergic vasculitis. However, histamine stimulates platelet aggregation through particular binding to the receptors of H_1_ antagonists [164].

#### 4.2.3. Phosphates (Polyphosphate, Pyrophosphate)

Phosphates (polyphosphate, pyrophosphate) are contained by normal human platelets, and they share biochemical and ultrastructural similarities with bacterial acidocalcisomes (documented with calcium- and proton-accumulating features) [165]. Polyphosphate is an important inductor of coagulation; thus, the possibility of neutralizing it could represent one molecular target against thrombosis (but without disrupting hemostasis) [166]. However, a potent inorganic pyrophosphate is present in δ-granules at a concentration of 1.4–3.1 nmol/10^8^ platelets and is not released by thrombin stimulation [167].

### 4.3. Ions

Dense granules also contain a high concentration of cations, particularly Ca^2+^ (but also Mg^2+^, K^+^, and P^+^ [168,169,170]. Similar to in other cells, Ca^2+^ is involved in the external discharge of granules. The release of granules seems to be Ca^2+^-dependent under specific circumstances (e.g., enzyme-induced sensitivity) [168]. Moreover, the aggregation of human blood platelets seems to be influenced by divalent cations (Ca^2+^, Mg^2+^) [171].

## 5. The Composition of the Platelet’s Lysosomes

Besides α-granules and δ-granules, like other cells, platelets contain lysosomes [172]. They are membrane-bound organelles that contain highly glycosylated proteins (enzymes) that are involved in the degradation of different constituents (proteins, carbohydrates, and phosphate esters) while protecting the lysosomal endomembrane [173]. The contents of these lysosomes are released from platelets after their activation, helping to achieve their functions, including when they are delivered in PRP.

## 6. Platelet T-Granules

T-granules represent a relatively recently described system of electron-dense tubules within platelets. T-granules consist of, inter alia, TLR9, VAMP7, and VAMP8, which are involved in the regulation of platelet distribution upon their activation [21]. Mammal platelets show (either human or murine) the positive expression of toll-like receptors (TLRs). Moreover, TLRs are upregulated during proplatelet production [174]. It was previously shown that the contact of platelets with collagen IV determines the overexpression of surface TLR9 and CD62P [175]. Moreover, T-granules exclusively contain protein disulfide isomerase (PDI), which is a major contributor to thrombus formation [176].

## 7. The Physiological Process of Skin Repair/Regeneration

The skin is a multifaceted organ, the largest of the human body. External threats to this protective barrier are represented by harmful UV radiation, intrusive pathogens, heat, and trauma. Notably, apart from providing a protective envelope, the skin is vital in safeguarding internal organs. Being the first line of defense against the external world, the skin is persistently at risk of damage, making the wound repair process indispensable. Gaining a deeper insight into the cellular and molecular dynamics of wound repair can pave the way for interventions that expedite the healing and regeneration process. This knowledge can be particularly beneficial for severe burn victims or amputees, especially when confronted with substantial tissue damage and scar formation.

Wound repair is an evolutionary process shared across species, encompassing stages that overlap in both time and space. These stages include inflammation, coagulation, and the dual process of cellular proliferation combined with extracellular matrix (ECM) restructuring [177,178]. Yet, the outcome of skin wound healing varies among different species. Some primitive vertebrates, such as zebrafish and certain amphibians like axolotls and xenopus, have the remarkable ability to regenerate their skin flawlessly. For instance, the full-thickness skin removal in these species is followed by the regeneration of the entire skin, together with its secretory structures [179,180]. Intriguingly, within this regenerative process, even the distinctive skin pigmentation patterns can be perfectly restored. Similarly, injured zebrafish skin not only reclaims its striped pigmentation but also regenerates the underlying fat cells and scales. It eventually results in normally structured and healed skin that is nearly identical to the previously un-lesioned skin [181].

While certain vertebrates demonstrate an impressive capability for skin regeneration, adult mammals, including humans, typically do not have such regenerative prowess. In most adult mammals, wounds often culminate in scar formation, usually characterized by skin appendages. While these scars serve the basic protective function of the skin, warding off infections and preventing dehydration, their presence is not entirely featured by benignity. Owing to their pronounced deviation from the native skin in appearance, scars, especially from severe burns or injuries, can lead to significant cosmetic and psychological distress, thereby affecting an individual’s overall quality of life.

The importance of skin appendages transcends mere aesthetics, playing pivotal roles in the skin’s biology and physiology. Nevertheless, epithelial skin appendages are sources of epidermal cells, contributing to the process of wound repair. Structures like hair follicles and sebaceous glands have roles beyond aesthetics, functioning either as a sensory element due to the special interactions they have with nerve endings, or as thermoregulatory units [182,183]. Consequently, the emergence of scars inhibits the full restoration of the skin’s multifaceted functions.

## 8. Cellular and Biochemical Milieu of Skin Repair/Regeneration Scene

Wound healing is a multi-step process, and it is crucial to grasp the initial cues that spark the cellular reaction in injured tissue. The activation of transcriptional systems is progressive. Consequently, the wound initially triggers transcription-independent pathways for immediate activation. Primary among these is Ca^2+^ waves, reactive oxygen species (ROS) gradients, and purinergic signals. Within the first moments following the injury, there is a surge in intracellular Ca^2+^ at the wound’s periphery, which then radiates towards its center [184].

Various signaling molecules, released by the damaged cells, play a pivotal role in attracting inflammatory cells to the wound site, neutrophils being the major cellular population. The involved signaling molecules, termed damage-associated molecular patterns (DAMPs), encompass a wide range of components such as DNA, peptides, components of the extracellular matrix (ECM), ATP, and uric acid. Hydrogen peroxide (H_2_O_2_) and lipid mediators, along with chemokines, are also released by the injured cells, further facilitating this recruitment [185]. Research involving multiple organisms has highlighted the importance of the swift generation of H_2_O_2_ at the wound site. Not only does this serve as a defense against potential infections, but it also propels the regeneration of keratinocytes, draws in neutrophils, and aids in the formation of new blood vessels [186].

### 8.1. Neutrophils

Neutrophils are only transient within the human dermis, and they originate from bone marrow promyelocytes. In the case of injury or infection, neutrophils are swiftly mobilized to act as the first line of defense. This recruitment is prompted by signals known as “find me” cues, which include DAMPs, H_2_O_2_, lipid mediators and chemokines emitted from affected areas [187]. Neutrophils are equipped with more than 30 different surface receptors, such as G-protein-coupled receptors (GPCRs), Fc receptors, integrins, and pattern recognition receptors, enabling them to sense these injury-induced signals. About 24 h after the injury, neutrophils account for approximately 50% of all cells within the wound site [188]. Once activated, these cells can release compounds that both prolong and amplify further neutrophil migration to the site [187]. To combat infectious agents, neutrophils employ various mechanisms like toxin granule release, initiating oxidative bursts, undergoing phagocytosis, and forming neutrophil extracellular traps (NETs).

During their maturation, neutrophils form distinct granules, each packed with specific agents designed for dedicated functions. Azurophilic (primary) granules are the first formed while the neutrophil is still maturing in the bone marrow and [189] are the latest to be secreted. Their primary role is intracellular bacterial destruction, merging their action to that of phagolysosomes. These granules contain components like myeloperoxidase, azurocidin, lysozyme, bacterial permeability-increasing protein, and several serine proteases, including cathepsin G, elastase, and protease 3 [190]. Subsequently, the secondary (or specific) granules are later formed, housing human cationic antimicrobial protein (hCAP-18), lactoferrin, matrix metalloproteinase 8 (MMP-8), and collagenase-2 [191]. Gelatinase is a marker for terminal neutrophil differentiation, and gelatinase granules are specialized for MMPs with gelatinase activity [192]. The final developed granules are secretory vesicles, which pack integrins, growth factors, and cytokine receptors for swift release from the cell [193].

Proteases, constituting a significant portion of the toxic granules in neutrophils, play a dual role in their activity. These enzymes are crucial for antimicrobial actions and for the degradation of the basement membrane and the extracellular matrix (ECM). This degradation facilitates neutrophils to cross from blood vessels into the affected tissue [194]. Additionally, proteases act as activators for matrix metalloproteinases (MMPs) and simultaneously inhibit protease inhibitors, intensifying the proteolytic activity. Studies involving elastase knockout mice have revealed a diminished neutrophil capability in bacterial clearance [195], highlighting the crucial role that elastase plays in the wound healing process. Nonetheless, an overproduction of proteolytic enzymes sourced from neutrophils, especially as observed in chronic wounds, can have detrimental effects. This excessive enzyme production can lead to the cleavage of growth factors, their respective receptors, and the ECM. Such cleavage impedes vital vascular activities, disrupts blood circulation, and inflicts tissue damage [196].

When activated, neutrophils also release neutrophil extracellular traps (NETs). These are intricate structures formed from chromatin fibers that spread into the extracellular domain. Laden with histones, cytosolic proteins, and proteases, NETs serve as a primary function of attracting and neutralizing foreign pathogens, including bacteria, fungi, and viruses. This extracellular trap mechanism is an essential part of the immune response, offering a unique way to combat invading microorganisms beyond traditional phagocytosis [197,198].

### 8.2. Macrophages

Macrophages are immune cells with a particular immunophenotype, being CD45-positive/CD11b-positive/CD66b-negative. Within a normal course of wound progression, the peak of the accumulation of macrophages within an injury site was 24–48 h [199]. For example, in young and healthy mice, wounds typically closed within 14 days. The macrophage concentration within the wound peaked around day 3, then reduced by day 5, and nearly returned to base levels by day 10. This macrophage surge can be attributed to both the proliferation of local tissue-resident macrophages and the recruitment of monocytes from bone marrow [200].

The exact role of tissue-resident macrophages during wound healing remains somewhat ambiguous. It is speculated that these macrophages, present in the skin since embryonic development, proliferate during wound healing. However, definitive evidence is still pending. Most of this research focuses on monocytes that differentiate into macrophages once inside the wound [201,202]. These monocytes are lured to the wound in reaction to degranulation events from platelets and mast cells, elevated levels of hypoxia-inducible factors, and the presence of chemokines like stromal-derived factor 1 (SDF1/CXCL12). Wound-residing macrophages also play a role in summoning more monocytes, intensifying the macrophage-mediated inflammatory response through the secretion of powerful chemoattractant(s), such as monocyte chemoattractant protein (MCP)-1 [203].

Macrophages hold significant importance in standard wound healing and tissue rejuvenation. In vivo experiments on mice, involving subjects deliberately depleted of macrophages, revealed delayed wound closure [204,205]. Such macrophage-deficient wounds manifested a compensatory neutrophil influx, coupled with a decline in angiogenesis, granulation tissue formation, collagen layering, and growth factor secretion [206,207]. On the other hand, artificially boosting the count of monocytes or macrophages in wounds can notably hasten the healing process in both typical and diabetic mice wounds [208]. Observations from other species align with these findings. In salamanders, a complete macrophage depletion post-limb amputation prevents limb regeneration. Remarkably, restoring the endogenous macrophage population post-amputation in these previously depleted salamanders can reinstate limb regeneration capabilities [209].

Macrophage malfunction is distinctly apparent in the compromised healing processes seen in diabetic wounds. Diabetic wounds manifest a temporal delay in the expression of chemokines essential for both monocyte attraction and macrophage stimulation [210]. This lag in macrophage recruitment results in postponed efferocytosis—the process by which dead or dying cells, such as neutrophils, as well as ECM and wound debris, are cleared away. Consequently, the transition to the proliferative phase of wound healing is delayed [211]. Over time, this leads to the establishment of a persistent inflammatory state, with both macrophages and apoptotic cells continuing to be dominant even during the remodeling phase of wound healing [212]. Furthermore, the macrophages present in diabetic wounds are not as efficient as in non-diabetic wounds. Their ability to release growth factors is hindered, and they are less effective in fostering neovascularization [213]. This compromised functionality further exacerbates the challenges associated with wound healing in diabetic patients.

### 8.3. Mast Cells

Mast cells play a pivotal role in skin defense, during the initial stages of wound healing. They release antimicrobial peptides that act as a protective barrier against potential skin infections [214,215,216]. Integral enzymes like chymase and tryptase, synthesized by mast cells, facilitate the breakdown of the biochemical components of the ECM. Moreover, these cells produce histamines and VEGF, which increase vascular permeability and facilitate neutrophil infiltration into the wound site [217,218]. It has been found that mast-cell-derived histamine actively promotes the proliferation of keratinocytes, with crucial implications in the wound re-epithelialization process [219]. Additionally, mast cell tryptase and histamine have been demonstrated to amplify fibroblast proliferation and boost collagen synthesis. This heightened activity is crucial in promoting and progressing wound contraction [220,221].

However, there is also a downside to this increased mast cell activity. Published data indicated that elevated numbers of mast cells have been linked to scarring and skin fibrosis [217]. Their impact on scar development was evaluated using a fetal wound healing model. According to this model, while wounds inflicted on mice on embryonic day 15 healed without forming scars, those on embryonic day 18 resulted in scarring. Intriguingly, introducing mast cell lysate into the day 15 wounds prompted a transition from scar-free healing to scar generation. In contrast, depleting the mast cells on embryonic day 18 led to diminished scar formation [222]. While these experiments underline the significant involvement of mast cells in scar development, the exact mechanisms remain to be fully elucidated. Additionally, the influence of mast cells on chronic wounds has not been extensively explored [223]. Preliminary findings suggest diminished mast cell numbers and reduced degranulation during diabetic wound healing. However, more comprehensive studies are needed to delineate their role in cases of compromised wound healing [224].

### 8.4. Langerhans Cells

Langerhans cells, resident in the epidermis, act as cellular sentinels, monitoring for pathogens and orchestrating contact hypersensitivity reactions [225]. Originating from early myeloid progenitors (EMPs) during embryogenesis, these cells migrate into the epidermis [226]. Throughout adulthood, their presence and density within the epidermis are predominantly governed by signaling from proximate keratinocytes [227]. When these cells recognize antigens, there is a downregulation of e-cadherin expression, which otherwise anchors them to keratinocytes [228]. This modulation facilitates their transit from the epidermis, through the dermis, culminating in their eventual arrival at the draining lymph nodes. Here, they play a seminal role in kindling a T-cell-driven adaptive immune response [226,229].

Apart from the resident dendritic cells (DCs), there is another variant, plasmacytoid dendritic cells (pDCs), which are not typically found in skin, in physiological conditions. However, these cells are ushered into the skin post-injury or during an infection [230]. After skin injury, the wound witnesses an immediate, albeit transient influx of pDCs. These pDCs, in reaction to self-nucleic acids liberated by the injured cells, become activated. This activation leads them to produce interferon (IFN)-α/β, facilitated by TLR7 and TLR9 receptors [230]. The experimental depletion of pDCs during the wound healing process has shown significant damping of the acute inflammatory cytokine response, leading to a lag in wound re-epithelialization [231].

### 8.5. Dendritic Epithelial T-Cells (DETCs)

Dendritic epithelial T-cells (DETCs) play a pivotal role in the immediate response following skin injury. When the skin is wounded, the affected keratinocytes exhibit an increase expression of specific ligands, notably SKINTs and CD100. This elevation in ligand expression triggers the activation of DETCs [232,233]. Within the first 24–48 h post-injury, there is a discernible transformation in the morphology of DETCs from a dendritic shape to a more rounded silhouette [234]. Upon activation, these DETCs become sources of various growth factors, e.g., keratinocyte growth factors (KGFs). Among these, KGF-1, KGF-2, and insulin growth factor-1 (IGF-1) stand out for their significant influence in boosting the proliferation of keratinocytes in the wound vicinity [235,236]. The crucial role of DETCs in wound healing can be emphasized by observing mice models. Mice that are devoid of DETCs demonstrate a marked delay in wound closure. This lag is characterized by diminished keratinocyte proliferation, postponed macrophage infiltration, and a reduced deposition of components of the ECM, such as hyaluronan [237].

Noteworthily, the process of wound healing undergoes alterations as organisms age. In aged mice, wound healing appears to be compromised, a phenomenon attributed to dysfunctional signaling dynamics between injured keratinocytes and DETCs [232]. Such observations highlight the critical role of DETCs and their interactions with keratinocytes in ensuring an effective wound healing response.

### 8.6. Invariant Natural Killer Cells (iNKTs)

Invariant natural killer cells (iNKTs) represent a unique subset of lymphocytes that simultaneously express markers indicative of both αβT-cells and NK cells. These cells play a multi-valent role in immunological responses, exerting regulatory control over allergic reactions, autoimmune conditions, and safeguarding the host against various pathogens [238,239]. One of the significant roles of iNKTs pertains to the process of acute wound healing, during which they produce IFN-γ, a cytokine with a crucial role in the inflammatory response. Experiments utilizing mice that lack iNKT cells reveal pronounced delays in wound closure. These delays are accompanied by a reduced deposition of collagen, diminished presence of α-smooth muscle actin—a marker for myofibroblast differentiation—and compromised neo-angiogenesis, or new blood vessel formation, within the wound [240]. Furthermore, iNKT cells serve a protective function, curbing prolonged neutrophil-driven inflammatory responses that could be deleterious to the healing process [241].

Turning to another crucial component of the immune system, T-cells, anomalies in their function have been associated with the development of skin fibrosis. In the context of human hypertrophic scars—a pathological, raised scar that may result from burn injuries—there is an increased presence of T-cells [242]. This interesting observation finds resonance in murine studies. These experiments indicate the fact that scar development is intimately tied to the activation of a Th2 CD4 T-cell response. This response is based on the release of specific interleukins, namely IL-4, IL-5, and IL-13 [243,244,245,246]. Considering the mechanistic aspects of scar development, heightened mechanical forces exerted on healing skin have been linked to the activation of T-cell pathways in hypertrophic scars [247]. This provides an interesting interplay between mechanical forces and immunological responses in the context of wound healing and scar formation.

## 9. Dermal TCs: A Distinct Cell Population with a Promising Skin Regenerative Potential

**Figure 2:**
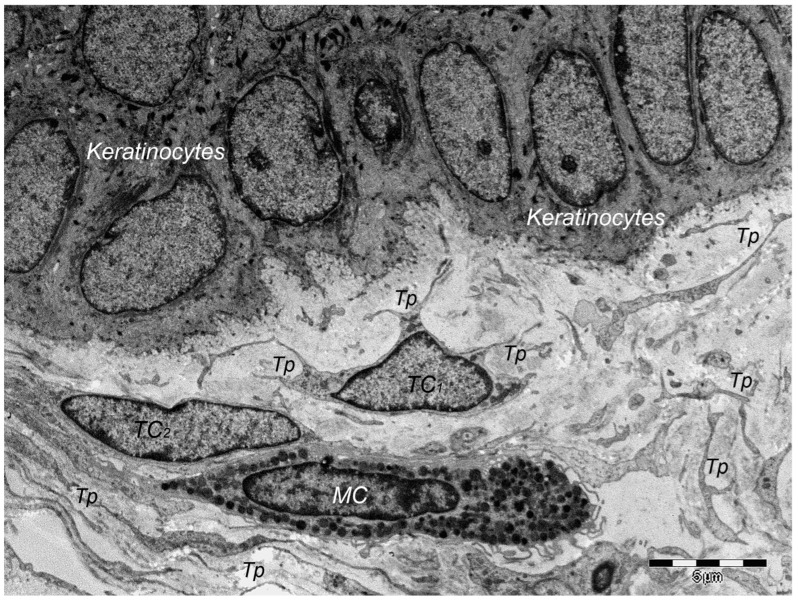
Skin; hair follicle (the outer sheath) and the surrounding dermis. Transmission electron microscopy. Two telocytes (TCs; TC1 and TC2) are located beneath the basement membrane. Within dermis, TCs are presenting cellular prolongations of uneven calibre—telopodes (Tps)—parallel to the basement membrane, and Tps are supplementary branched. Tps are long cellular structures, their entire length being partially intercepted by this plane of section. Supplementary, TC2 (through its Tps) is establishing close contact with a mast cell (MC).

**Figure 3:**
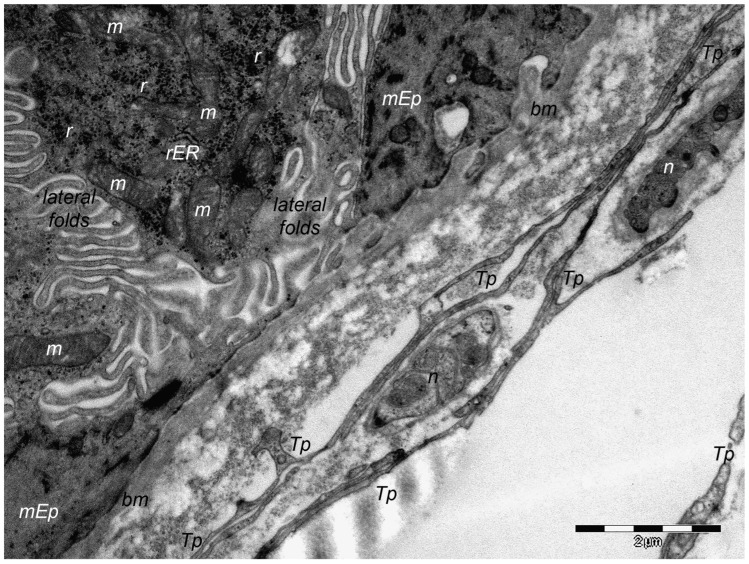
Skin, eccrine sweat gland. Transmission electron microscopy. Neighbouring telocytes (cellular bodies were not encompassed into the microscopic field) are establishing cell contacts by their cellular processes—telopodes (Tps). Moreover, the Tps are branching and enwrapping a nerve ending (n), thus repeating structural positioning motif found in different location of the skin, but also within interstitial space of other organs. Telocytes locations are outside the basal lamina (bm) of the eccrine sweat gland. Above the basal lamina, the basal pole of the clear epithelial secretory cells extensively containing mitochondria (m), ribosomes (r) and elements of the rough endoplasmic reticulum (rER). Aside the clear epithelial secretory cell, two myoepithelial cells (mEp) are visible. Supplementary, the creases of the basal-lateral sides plasma membranes are extensively amplified, creating a labyrinthine structure specific for the clear epithelial secretory cells.

**Figure 4:**
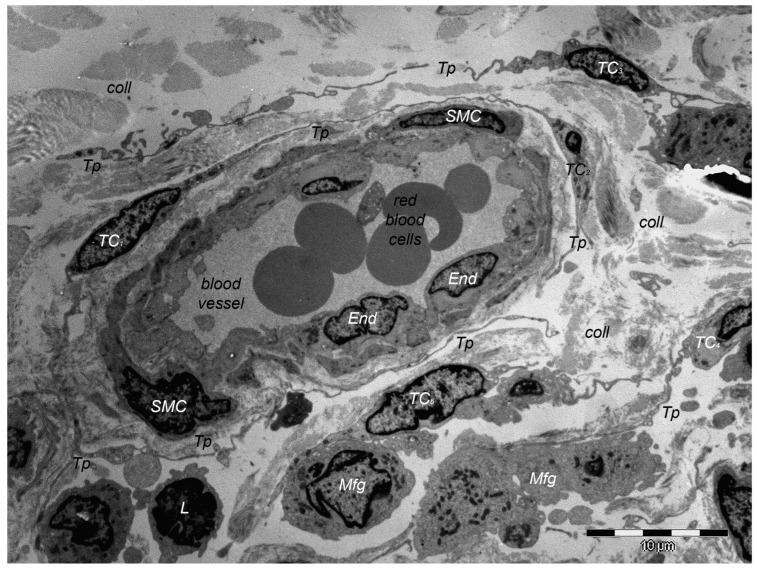
Reticular dermis. Transmission electron microscopy. Telocytes (TCs_1-5_) and their telopodes (Tps) are surrounding closely a blood vessel. TCs are in the range of molecular contacts to ab luminal surface of the endothelial cells (End) and smooth muscle cells (SMC). Moreover, Tps most probably belonging to other TCs (not encompassed by this plane of section) are concentrically disposed and seem to be involved into an external network of TCs. They are in contact with other types of interstitial cells—lymphocyte (L), macrophages—Mfg. Various packages of collagen fibres (coll) of different thickness are irregularly disposed within reticular dermis.

Over one decade ago, a distinct type of interstitial cell was initially documented in the stroma of few different peri-digestive organs [248] and were named telocytes (TCs) due to their morphology and location within tissues. Morphologically, their most prominent trait is the presence of very long cellular prolongations featured by a particular long-lump aspect—termed telopodes (Tps). From the peri-digestive compartment, their presence was afterwards documented in many different other organs from different species, including the (human) skin [249,250,251,252,253,254,255,256,257,258,259,260,261,262,263,264,265,266,267,268,269,270,271,272,273,274,275]. Interestingly, in all these locations, TCs display the same peculiar structural and ultrastructural features, as described in other organs, only with some minor variation in terms of their immunohistochemical phenotype [251,276,277,278,279,280]. Since their presence always features the stroma (connective tissue) of organs, it was understandable to find TCs within skin dermal layers [276,281]. In this location, TCs are grossly distributed in the deeper reticular dermis, in close vicinity to hair follicles strictly boarding the glassy membrane of the hair follicle (Figure 2), sweat glands (Figure 3), or sebaceous glands [282]. TCs are less dense in the superficial papillary dermis. Powerful and complex scanning electron microscopy techniques gave valuable conformational details about TCs and the spatial conformation of their Tps (as they are mostly veil-like structures rather than thread-like cellular prolongations [282]. Also, these techniques proved the complex three-dimensional disposal of dermal Tps (Figure 4), and their disposal in a network involving other TCs (by their Tps) and/or other types of interstitial cells, nerve cells, endothelial cells, or even immune cells [283,284].

However, in terms of ultrastructural phenotype, dermal TCs show a particular double-positive expression for CD34 and PDFGRα. Moreover, in comparison with the connective tissue, mostly present in interstitial cells—the fibroblasts (Fbs)—TCs possess a characteristic positive expression for epithelial-derived neutrophil-activating peptide-78 (ENA-78) and granulocyte chemotactic protein 2 (GCP-2) [10], molecules also found within the platelets’ α-granules. Contrarily, dermal Fbs have a characteristic positive expression for CD90 (procollagen type I), and supplementarily, ELISA testing has confirmed their differential expression for 37 other cytokines, which are more highly expressed than in TCs (IL 5, MCP-3, MCP-4, MIP-3, angiogenin, and thrombopoietin) than Fbs [11].

**Figure 5:**
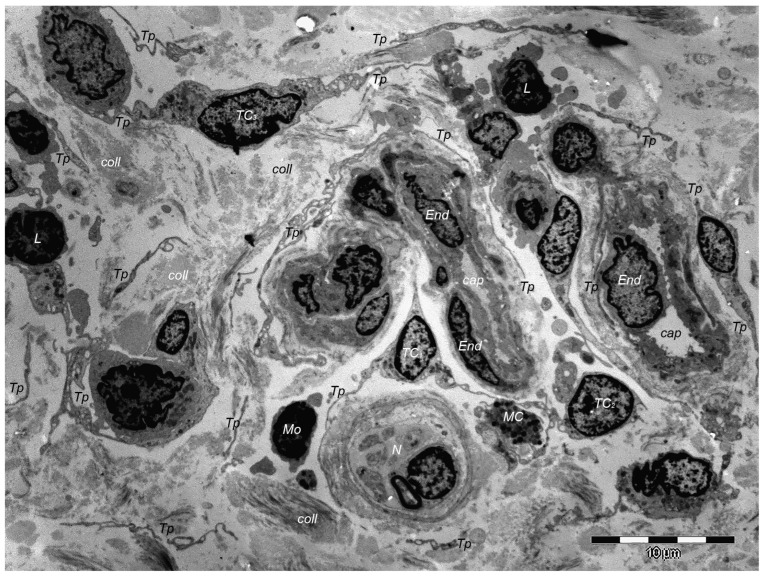
Dermis. Transmission electron microscopy. A typical ultrastructural aspect of a dermis. Telocytes are displaying the well acknowledged morphology: a small cell body but long, tortuous, and lumpy cellular prolongations—telopodes (Tps). By Tps, TCs are involved in close vicinity with capillaries (cap), and different types of immune cells, e.g., mast cells (MC) and monocytes (Mo). Fragments of Tps (belonging to other TCs outside the plane of section) are apparently randomly disposed, highlighting the acknowledged distribution of TCs in 3-dimensional networks, thus supporting their attributed presumptive roles. End—endothelial cells; N—nerve ending; coll—collagen fibers.

Of course, corroborating structural data and immunohistochemical data many presumptive roles were suggested for dermal and non-dermal TCs, including intercellular signaling [285], interstitial homeostasis [11], vascular homeostasis [284], (neo)angiogenesis [12], wound healing [286]. Roles of nursing cells either in epithelial stem cells or implications in skin immune physiopathology (interactions with mast cells) are suggested, considering the cytokine/immune profile of TCs. Moreover, these hypotheses consider TC networking within normal tissue and, moreover, TC-specific localization within reticular dermis, in close vicinity to skin adnexa, blood vessels, and nerve endings (Figure 5). TCs’ paracrine secretion (shed vesicles) suggests roles for TCs in either controlling or modulating dermal Fbs’ activity or phenotype [276], or in organizing the dermal connective tissue environment [287]. Additionally, the positive expression of TCs for several angiogenic factors (either in skin, or in other organs [10,288]) strongly suggests TCs’ roles in (neo)angiogenesis, additionally documented by ultrastructural studies [12,289,290].

## 10. Dermal Telocytes’ Involvement in the Course of a Few Dermatologic Pathologies

The presence and distribution of TCs within the dermis is influenced either by or a few studied physiological or pathological conditions of the skin [282]. In all pathological conditions in which TCs were studied in terms of their distribution and density, these parameters were documented as being affected differently by various skin conditions and their different levels of severity [276]. Previously documented data on various dermatological disorders indicated a series of changes in dermal TCs either in terms of dermal distribution, structural changes, or participation to lesion progression/remission [291]. Thus, the abnormal accumulation of dermal collagen (a structural feature of scleroderma), particularly collagen II and the reticular fibers (collagen III) along mucoid oedema and panniculitis (that are structurally also featuring scleroderma) produces separation of TCs in their networks, disarranging this supra-cellular structure, and individualizes affected TCs, with effects on the architecture of the normal dermal tissue [292,293]. The TC density reduction is initially seen in the superficial dermis, but the reticular dermis follows these changes closely [293]. Within fibrotic masses or enwrapped in edematous areas in scleroderma lesion skin, TCs display degenerative ultrastructural features, having enlarged cell shapes, also with enlarged Tps and distended mitochondria with scarce cristae within a dense cytoplasm. Moreover, the cytoplasm of affected TCs also contains vacuoles and lipofuscin bodies [287]. Within scleroderma TCs, degenerative processes advance hypoxically along the disruption of the TC network, and along with the fibrotic process, simultaneously altering the extracellular matrix. However, interesting ultrastructural details showed no TCs in the vicinity of occluded blood vessels, but rather TCs of normal morphology surrounding blood vessels with an opened lumen and thickened basement membrane [287]. In scleroderma skin, numerically reduced TCs are mainly found around skin adnexa and nerve fibers [282]. Moreover, in experimental bleomycin-induced scleroderma, the dermis shows a reduced density of TCs, also with disintegration of their ultrastructure. The nuclear fragmentation is more prominent, with the condensation of chromatin in apoptotic fashion [292]. However, the documented TC changes in scleroderma and the whole cellular and molecular tissue milieu are still a debate, since there are different opinions about what came first [11]. Some opinions advocate for TC changes being secondary to a hypoxic microenvironment, and others for the accumulation of large deposits of collagens due to TCs ultrastructural changes [293].

Psoriasis, an immune inflammation of the skin, is featured by (ultra)structural changes of TCs, including ultrastructural dimorphisms of the cellular bodies with cytoplasm fragmentation and cell membrane shatters with nuclear exclusion). Moreover, Tps were documented with serial fragmentations [282]. Disruption of TCs network integrity with ultrastructural changes of TCs seemed to coexist with serial changes in the activity of antigen presenting cells—the Langerhans cells—and their presence and increased activity below the dermal-epidermal junction, within the dermis [10].

TCs presence is influenced either by neoplastic pathology, since in basal and squamous cell carcinoma they establish preferentially heterocellular junctions or contain cytoplasmic dense electron plaques [285].

## 11. Variable Results of PRP on Treated Skin

However, despite the growing popularity of PRP administration, the effects of PRP administration in different dermatological conditions could be highly variable, being influenced by numerous unpredictable factors. These factors could be related to patients’ unique biological characteristics, but they could also be dependent to specific protocols used in the preparation and application of PRP. Thus, several factors are contributing to the variability in outcomes, and these could fall into two major categories—patient-related factors and PRP-product-related factors [294,295,296,297,298,299]:

The age of the patients represents a condition for PRP therapy outcomes. It is well known that younger patients have a more robust and reliable repairing process compared to older individuals. It is acknowledged for different organs that the regenerative capacity is decreasing with age, and this can alco affect the results of the PRP treatments on the skin.

Different skin types respond differently to the PRP treatments, depending on the structure of involved skin (thin or thick).

The severity of the affected skin could affect the outcome of PRP application. There are many cases of skin lesions in which additional alternative treatments could be necessary to deliver good results in PRP treated patients.

The health status of the treated patients could interfere with the results of PRP treatments, usually the healthy individuals have better responses to the PRP treatment.

Products containing a higher concentration of platelets (containing their corresponding growth factors and cytokines) are associated with better results. However, excessively highly concentrated products might not necessarily yield better results.

Different protocols used for the preparation of PRP products deliver different concentrated platelet products. However, it is well known that the speed and duration of centrifuge spinning can influence the quality of PRP products. Thus, consequently, different methods of PRP preparation and concentration deliver different results.

The technique of administration (injection, micro-needling, etc.), single or in combination with other techniques or treatments, is also influencing the results. Moreover, the anatomical region, the depth and the pattern of injection represent some critical factors for PRP outcomes.

PRP treatments offer a promising and smart approach to skin rejuvenation, employing, leveraging, and eliciting the body’s natural resources involved in the healing processes. However, the effects of PRP on the skin are variable and frequently biased by multiple factors. As research progresses and the protocols become more standardized, the predictability and efficacy of PRP therapy are both likely to improve, making PRP treatments more reliable options for skin rejuvenation.

The previous published results offer a mixed view of PRP’s efficacy in skin rejuvenation. There are research groups showing that PRP therapies are showing promising results [300,301], improving the skin texture, rhytids and fine lines, the general aspect and skin luminosity of the study participants. Conversely, other studies have reported minimal to no significant improvements, indicating the variable nature of PRP therapy [302,303]. However, these variances in reports indicate a stringent need for standardizing the protocols and for identifying those factors that could optimize the PRP efficacy [304].

## 12. PRP Treatment Limitations, Contraindications, and Practical Considerations

Supplementary, PRP therapy has several limitations that can affect its efficacy and applicability. As variable outcomes are resulting from patient-specific factors (e.g., health status, severity of the dermatological condition to be treated), or PRP preparation (which into the lack of standardization leads to a great variability that can affect treatments outcome. A thorough evaluation of the patient’s skin condition(s), medical history, and individual characteristics will help determine whether PRP represents an appropriate treatment option. However, for the patients who are considering PRP therapy for skin rejuvenation, it would be preferable and essential to have realistic expectations to the treatments and to understand the potential variability in outcomes.

The clinicians should adhere to evidence-based protocols for platelet product preparation and administration to maximize positive outcomes [298,305]. This includes using standardized centrifugation techniques that ensure an optimal platelet concentration, and the employment of precise injection methods. Moreover, the combination of PRP with other treatments, such as laser therapy or microneedling, may enhance the results for selected patients [306,307].

On the other hand, there are some specific situations where the procedure should not be used due to its unfavorable potential for the patient. Usually, the PRP therapy contraindications can be broadly classified into absolute and relative contraindications [298,308,309].

The absolute contraindications could include the Platelet Dysfunction Syndromes (e.g., the Glanzmann’s thrombasthenia) that make PRP ineffective due to functional impairment of the platelets, the severe thrombocytopenia which is featured by the significantly depleted number of platelets, that making thrombocytes numerically incompatible with an effective treatment [310,311]. Supplementary, the acute infections (or sepsis) will make PRP treatments inadvisable due to the risk of infections spreading or exacerbation of the patient’s condition(s). Moreover, the anticoagulant therapy using different molecules (e.g., heparin, warfarin, etc.) may increase susceptibility to bleeding and bruising at the injection site [312,313]. Different neoplasms, especially the hematologic malignancies, disallow PRP since theoretically, containing growth factors, PRP could potentially accelerate the cancer cell growth [314].

Some of relative contraindications include the chronic infections that could pose a risk (thus a detailed evaluation could be necessary), or pregnancy/breastfeeding that, due to limited data, suggest caution. On the other hand, autoimmune diseases (e.g., lupus, dermatomyositis or rheumatoid arthritis) could be accompanied by paradoxical response to PRP administration [315]. The (uncontrolled) diabetes could interfere with the recovery after PRP administration, impairing the wound healing and the immune response, thus favoring the risk of complications [316,317]. Also, since corticosteroids are interfering with the efficacy of PRP treatments, steroid injections should be avoided several weeks before such treatments [318].

## 13. Limitations of PRP Therapy

In dermatology, and in aesthetic medicine, PRP therapy has gained popularity for treating various conditions such as hair loss, acne scars, and skin rejuvenation. However, it has several limitations considering that the results can be inconsistent, varying significantly between patients due to individual differences in platelet concentration and overall health. Additionally, PRP therapy typically requires multiple serial sessions that can last several months and can be time-consuming and costly.

The PRP preparation and administration process also lack standardization, leading to variability in outcomes. To date, there is no universally accepted protocol for PRP products preparation and administration [319]. Thus, differences in centrifugation techniques, platelet concentrations, and injection methods can result in inconsistent results. The optimal dosing and the frequency of PRP administration are not well standardized and established. While some conditions may require a single treatment, others may benefit from multiple sessions, and the ideal interval between treatments is not well defined [320].

Moreover, PRP therapy may not be effective for advanced cases of hair loss or severe skin conditions, limiting its applicability. The placebo effect can play a significant role in perceived improvements following PRP therapy. Blinded, controlled studies are necessary to distinguish actual therapeutic benefits from placebo responses [321,322].

While PRP therapy shows promise and exhibits excellent results (at least in regenerative dermatology), many studies suffer from small sample sizes, lack of control groups, and short follow-up periods [323]. More robust, high-quality research is needed to confirm its efficacy and safety across various applications [324]. Perhaps, long-term exhaustive studies are needed to fully understand the efficacy and safety of PRP therapy in dermatology, as current evidence is still somewhat limited and not universally accepted [325].

Although PRP is derived from the patient’s own blood, there is still a risk of infection at the injection site if proper sterile techniques are not followed [315]. Incorrect injection technique can lead to tissue damage, nerve injury, or other complications, highlighting the importance of skilled practitioners [326]. The procedure can also cause temporary side effects such as pain, swelling, and bruising at the injection site. While typically temporary, these symptoms can be distressing for patients [327].

Regarding the economic aspects of these procedures, PRP therapy can be expensive, as it involves specialized equipment and multiple clinic visits. The cost can be prohibitive for many patients, especially since it is often not covered by insurance. Thus, in many situations the access to PRP therapy may be limited in certain states, depending on their region or healthcare laws and rigors, restricting its availability to patients who might benefit from it [328,329]. However, patients may receive different levels of care depending on where they seek treatment. The promotion of PRP therapy for unproven or off-label uses raises ethical concerns. Clinicians must balance enthusiasm for new treatments with a commitment to evidence-based practice [330]. The regulation of PRP therapy varies by country and region, leading to inconsistent standards of practice and quality control [331].

## 14. Conclusions and Perspectives

PRP therapy represents a promising treatment modality across various medical fields, offering potential benefits for patients with a range of conditions. However, its use is not without contraindications and limitations. Absolute contraindications such as platelet dysfunction syndromes, severe thrombocytopenia, active infections, active cancer, and anticoagulant therapy must be carefully considered to avoid adverse outcomes. Relative contraindications, including chronic infections, autoimmune diseases, uncontrolled diabetes, pregnancy, breastfeeding, and recent corticosteroid injections, require thorough evaluation and patient-specific considerations.

The limitations of PRP therapy, including variable outcomes, lack of standardization, insufficient high-quality evidence, cost, accessibility, procedure-related risks, and regulatory and ethical concerns, must be addressed to optimize its use and ensure patient safety. Continued research and clinical trials are essential to establish standardized protocols, determine optimal treatment regimens, and confirm the efficacy and safety of PRP therapy across its various applications. By recognizing and addressing these challenges, healthcare providers can better navigate the complexities of PRP treatment and enhance patient outcomes.

On the other hand, currently the advanced therapy medicinal products (ATMPs) represent the cutting-edge medical treatments that include gene therapies, cell therapies, and tissue-engineered products [332]. These technically refined and more powerful treatments offer potential cures for complex diseases by repairing, replacing, or regenerating human cells, tissues, or genes [333]. For regulatory reasons, PRP is not considered an ATMP [334]. Both PRP therapy and ATMPs represent significant advancements in personalized and regenerative medicine, offering innovative solutions to previously challenging medical conditions.

Presently, the field of PRP therapy is evolving, with ongoing research aimed at better understanding its mechanisms and optimizing its use in dermatology. Future studies are needed to establish standardized protocols and identify biomarkers that predict patient response to PRP. Additionally, advancements in technology may lead to more refined PRP formulations and delivery methods, further improving the efficacy and consistency of this treatment.

Considering all mentioned regarding PRP and interstitial TCs, further perspectives could be hypothesized in regard with TCs roles and their regenerative potential. TCs are a distinctive population of resident cells with particular relations with dermal structural elements and elements of the epidermal stem cell niche.

To date, a broad range of dermatologic therapies have reported a series of good results in skin resurfacing/rejuvenation/repair after minimally invasive procedures. Usually, producing inflammation within the dermis (by different means) aims to to finally produce collagen and other dermal structural elements that could repair a skin defect.

Currently, there is also a lack of detailed studies of the cellular/molecular milieu elicited by PRP administration, moreover from the perspective of dermal TCs. Additionally, a cytochemical and physical collaboration between TCs and stem cells with implications in tissue regeneration in other organs (e.g., heart, lung, etc.) was indicated in previous published results [11]. To date, there are so many microscopical and biochemical confirmations that TCs represent a dermal resident cell population that is immunophenotypically and ultrastructurally distinct and being totally different from any other known dermal resident cell population. Considering the gross implications in skin homeostasis and skin pathology, the study of its behavior in reparatory/regeneration instances is an intriguing way of research. It comes naturally to investigate the presumptive ways of managing dermal TC distribution/density that could help achieve a swift and natural recovery of skin lesions.

According to published data, there is a great body of evidence indicating the regenerative potential of TCs, and their presumptive roles in the structural and ultrastructural rehabilitation of damaged tissue. There is also a great expectation for these cells with respect to regenerative medicine or tissue renewal. Thus, it is tempting to postulate implications of dermal TCs in the renewal/repair/regeneration/remodeling development after administering PRP. Thus, we may perceive PRP (only) or combined with other regeneration/renewal procedures as promising candidates with stunning potential for replacing conventional therapeutic approaches [328].

However, experimental studies on the heart after experimental acute myocardial infarction indicated the important involvement of TCs in lesion recovery within the border zone of myocardial infarction [12,292]. Considering these results, and the entire sequence of tissue repair, it would be unimaginative and prosaic to consider fibroblasts as the only key players in tissue regeneration.

Hitherto, there are few studies conducted on different platelet concentration derivatives: plasma, PRP, PRP-derived exosomes (PRP-Exos)/PRP-derived extracellular vesicles (PRP-EVs), and platelet-poor plasma (PPP) [335]. As we previously mentioned, the administration of PRP provides the interior of the tissue a higher concentration of GFs with angiogenic, mitogenic, and anabolic properties, which represents a routine, alternative, less invasive treatment [303]. To date, many studies have involved the administration of PRP to follow-up patients at different time intervals—from 4 weeks to 4 years [336]. All these studies indicated a great potential of PRP in treating various structural alterations of skin integrity and normal structure. Using the administration of the biologically active molecules from the plasma secretome leads to new approaches for wound closures [337]. The wound healing studies using PRP-EV indicated safety for usage in humans but no net difference between PRP-EV treated wounds and placebo [338].

PPP usually encompasses plasma that is normally discarded after the preparation of PRP. PPP is autologous plasma with a low platelet concentration that can act as a sealant and scaffold for those migrating cells involved in wound healing and tissue regeneration [339]. However, it has been proven to contain great amounts of various bioactive proteins (fibrinogen, VEGF, PDGF, SDF-1α, etc.) [340]. The studies on PPP have demonstrated that it is a good hemostatic agent for wounds, but its efficacy in regeneration should be precisely documented by further studies [336,341].

Currently, clear detailed structural and ultrastructural studies on mechanisms induced by PRP are rare, and there is a persisting vagueness in the presented data. Further studies on PRP cellular phenomena with respect to TCs’ presence within the dermis that are based on strong microscopical evidence could facilitate unique, valuable, and challenging research on the ultrastructure of the reparatory cellular environment provided by the administration of PRP.

## Figures and Tables

**Table 1:** The composition of the platelets’ α-granules.

**GROWTH FACTORS**	Platelet-derived growth factor (PDGF), AA-AB-BB
	Transforming growth factor β (TGFβ)
	Vascular endothelial growth factor (VEGF)
	Epidermal growth factor (EGF)
	Fibroblast growth factor (FGF)
	Connective tissue growth factor (CTGF)
	Insulin-like growth factor (IGF-1)
	Hepatocyte growth factor (HGF)
	Keratinocyte growth factor/fibroblast growth factor-7 (KGF/FGF-7)
	Angiopoietin-1 (ANG-1)
**CHEMOKINES**	Growth-regulated protein alpha (CXCL1/GRO α/KC/CINC-1)
	Platelet factor 4/chemokine (C-X-C motif) ligand 4 (PF4/CXCL4)
	Epithelial-cell-derived neutrophil-activating peptide-78 (ENA-78)/C-X-C motif chemokine 5 (CXCL5)
	C-X-C motif chemokine 7 (CXCL7)/Neutrophil Activating Peptide 2 (NAP-2)
	C-X-C motif chemokine 8 (CXCL8)/interleukin-8 (IL-8)
	Stromal-cell-derived factor-1 α/C-X-C motif chemokine 12 (SDF-1α/CXCL12)
	Monocyte chemoattractant protein (MCP-1)/Chemokine C-C ligand—2 (CCL2)
	Macrophage inflammatory protein-1α (MIP-1α)/Chemokine C-C ligand—3 (CCL3)
	Chemokine C-C ligand—5 (CCL5)/regulated on activation, normal T-cell expressed and secreted (RANTES)
**ADHESION MOLECULES**	Fibrinogen
	Thrombospondin
	the von Willebrand factor (vWF)
**INTEGRAL MEMBRANE PROTEINS**	Integrin αIIbβ3
	Glycoprotein (GP) Ib-IX-V complex
	Triggering receptor expressed on myeloid cells (TREM)-like transcript 1 (TLT-1)
	P-selectin
**COAGULANTS AND ANTICOAGULANTS**	Platelet factor V
	Factor IX (Christmas factor)
	Factor XIII
	Growth arrest specific-6 (Gas6)
**ANTIMICROBIAL AGENTS**	
**INFLAMMATORY/IMMUNE AGENTS**	

**Table 2:** The composition of the platelets’ δ-granules.

**Nucleotides**	Adenosine triphosphate (ATP)
	Adenosine diphosphate (ADP)
	Cyclic adenosine monophosphate (cAMP)
	Uridine triphosphate (UTP)
	Guanosine-5′-triphosphate (GTP)
**Bioactive amines**	Serotonin (5-hydroxytryptamine)
	Histamine
	Phosphates (polyphosphate, pyrophosphate)
**Ions**	Ca^2+^, Mg^2+^, K^+^, P^+^

## Data Availability

The data that support the findings of this study are available on request from the corresponding authors, C.G.M. and M.E.H.
